# The Surgical, Mechanical, and Medical Treatment of the Teeth, &c.

**Published:** 1846-10-01

**Authors:** 


### The Surgical, Mechanical, and Medical Treatment of the Teeth, &c. By *James Robinson.* Pp. xx. 320. London, 1846.

There is not, we fearlessly assert, in the whole range of medical practice, no, not even including all the absurdities, and still more, the wilful falsehoods, of Homœopathy, of Hydropathy, of quackery in all its forms, from the last edition of the advertising and puffing book of the regular physician, to the last advertisement of Morrison's pills, or Holloway's ointment, any one branch or element which offers more startling anomalies or more atrocious examples of lying empiricism than the present practice of Dental Surgery. Let any one who takes umbrage at this assertion, or who feels a consciousness, although even himself a Dentist, that he does not deserve to be included in this disgraceful category, and there are many such, look at the advertising columns of the Times or the Chronicle, the Satirist or the Age, or let him order from across the Atlantic the last or any other number of Stockton's Dental Intelligencer (we believe this is the title of the work), and we ask for no further apology for our unhesitating and contemptuous sentence. The present state of the profession (the trade we had better say) of the Dentist, is one of the foulest blots in the page of medical history. In a branch of medical and surgical practice, which ought to be associated with a good general knowledge of the principles of physiology and of pathology, which can only be successfully and honorably followed by one whose professional education has been that of a physician and surgeon, whose information is only limited by the extent of the present improved teaching of our schools, and the general practice of our hospitals, we find that every Jew mechanic, who, from want of character or other not less disgraceful cause, fails in his trade—every charlatan who makes up for want of real knowledge of the profession by the most impudent pretension—every unhappy student who is plucked at the College or the Hall, considers himself fully competent to fleece the public in the character of a Dentist, and to practise, without knowledge sufficient to treat safely a whitlow or a cholic, a branch of the profession which includes as numerous and important a class of obscure sympathies and severe and even dangerous consequences, as those which are associated with any organ or system of organs in the human body.

Whence arises this shameful opprobrium? What is there essential to the practice of Dental Surgery that involves an anomaly so disgraceful, and so universal? Is there any one circumstance necessarily associated with this branch of the healing art, to which can in any way be traced this blight that hangs over it, and taints every one belonging to it with a portion of its evil? We believe that it is not difficult to discover its cause, in the universal association of the art of "*Dentistry*" (we rejoice in this vulgar Americanism as so appropriate to the case) with Dental Surgery. It is, we feel convinced, this and this alone, that has reduced dental surgery to the level of a mechanical trade, and never can it rise to take its legitimate rank with other branches of the profession of medicine and surgery, until this ill-sorted union is dissolved. It may perhaps be urged that it is impossible to sever the two. That there is not sufficient discrimination in the public mind to enable the mass of society to distinguish between the mere Dentist and the Dental Surgeon—that the association of the two has now become so universal that it would be a mere act of Quixotism in any individual to attempt the practice of one without the other. We have only to answer to this, that it has never yet been fairly tried, and, at all events, we feel that, until it is tried, no one has a right to complain of the position to which the professional dentist has reduced himself in the ranks of the profession.

We believe, however, that it may be done. An individual, perhaps, could do but little; and it would probably be ruinous to his practice, or at least temporarily injurious, for him to attempt it alone. But surely, if the educated members of the profession, those who have become recognized and authorized practitioners of surgery, by the acquisition of a diploma from either of the Colleges of Surgeons, or other similar body—the graduates, so to speak, in the profession—if these would but unite to throw off from themselves, as by one act, an opprobrium under which, we doubt not, many of them feel oppressed and degraded, the voice of the intelligent public would sanction the effort, and their confidence would reward an act of courage and honour so creditable to those who effected it, and so unspeakably beneficial to the public themselves.

This is not however the place, nor have we room, to enter at large into the details of any feasible plan for effecting so great an object. The hint may be of use—and heartily do we wish that any representation of ours could so rouse the respectable portion of this degraded branch of their profession, to a sense of the degradation which they are themselves perpetuating by every act of tooth-making, as should make their disgrace too heavy for them to bear, and force them to cast it off as an unworthy appendage to the legitimate and honorable practice of their profession.

The above remarks will at once evince to our readers, that the book before us will find but little favour at our hands, considered as a professional work. Its whole plan and scope involves the very union which we have been deprecating; and the very title has the anomalous arrangement of the *mechanical* interposed between the surgical and the medical treatment of the teeth; and, as if this were not a sufficient indication of the prominence given to the manufacturing department, we find in addition the words, "including dental mechanics."

Our duty as honest reviewers will not allow of our taking a partial view of any professional work which comes before us in our official capacity. Private respect for persons may sometimes render that duty very painful; but in proportion to the difficulty of being impartial is its necessity increased. We proceed, therefore, to examine what is the situation which this work ought to occupy in the estimation of the profession, with the determination to "nothing extenuate nor set down aught in malice."

The first impression made on our mind on opening the book is, that the author, from some cause, not perhaps very difficult to be understood, is deeply enamoured of the American system of dental practice. As he identifies himself *in limine* with our excellent friends across the Atlantic, as he is an "Honorary Doctor (!) of Dental Surgery of the Baltimore College of Dental Surgeons," (see Title), as he is, moreover, we believe, the accredited agent of a certain American periodical, before alluded to, we shall perhaps be excused if we consider this close association as some indication of the professional tendencies of our author. Now we are extremely willing to give all due credit to the American *Dentists,* as mere Dentists, as practitioners of what they so felicitously term "Dentistry"—a term which, by the way, Mr. Robinson has adopted and continually employs. We have been assured by those who know much of these matters, that they are extremely adroit at filling hollow teeth with gold (and, *par parenthèse,* it may be added that these are not the only hollows which they contrive to fill with the same precious metal), that they are clever at managing the arrangement of irregularities in the growth and position of teeth, and that the work which they exhibit in the construction of artificial teeth is something quite extraordinary: but what are their claims to be considered as SURGEONS—what their knowledge of the real principles of the treatment of disease,—what their physiology, their pathology, their diagnosis of associated and sympathetic disorders? We intend, on some future occasion, to enter at length into the discussion of these questions, not only with respect to American "*Dentistry,*" but with reference also to the existing state of this branch of the profession generally. At present we shall confine ourselves to such incidental examples as may come before us in our review of the present work; premising, however, that whatever may be our judgment as to the general state of the profession in America, we are willing to grant that there are some striking exceptions to the otherwise universal censure to which we sincerely believe them to be obnoxious, as mere mechanicians.

The title of the very first Chapter of the book before us, sufficiently exemplifies its real scope. It is entitled "The History of the Dental *Art*;" and the first words of this chapter are, "the origin of medicine, like that of many other *Arts*, is involved," &c. Now here is the fatal mistake which Dentists are very apt to commit. It is all very well for them to look upon their trade as a mere *art*—they have made it so, as a body, by the very mixture of practice which we have been deprecating;—but inasmuch as medicine is to be considered as something more than an art, so ought also dental surgery and dental medicine to be placed on the footing of a science, and its practice to be kept on a professional basis. We will, however, dwell no longer on this matter at present.

In the Chapter in question, we have an amusing digest of the ancient history of the treatment of the teeth, from the earliest ages; and the author has the following pertinent remark on the reasons which would naturally lead the Egyptians to take the lead in the supply of lost teeth by artificial means. "The significance of these organs,—to say nothing of their ornamental or useful functions,—was acknowledged in a remarkable manner by the ancient Egyptians, so that one of their most severe and infamous punishments consisted in the abstraction of a front tooth." "The loss of a front tooth, whether by disease or not, would naturally, under the circumstances of Egyptian law, give rise to unpleasant suspicion, and every exertion might be expected to be made to supply the deficiency. Accordingly, Belzoni and others have discovered artificial teeth in the Sarcophagi of the ancient Egyptians. These, it is true, are rudely made, and, from being of wood, are ill adapted for performing mastication, &c." (p. 8.)

This first Chapter closes with a comparison between the merits of English and American Dentists, a comparison no less invidious in its depreciation of the former than it is undeserved, if we exclude the numerous quacks and advertisers of our country from the list. Let us first see what he says of the English authors on this subject.

"About this period, the famous John Hunter turned his attention to the subject, and presented the world with his 'Natural History of the Teeth;' a production which, while it enlarged the sphere of dental knowledge, piqued the pride and roused the ambition of the English practitioners of the art.

"The inaugural Dissertation on the Structure of the Teeth of man and animals, published in 1798 by Robert Blake, gives evidence of the rapid strides that had been made in the anatomy and physiology of the teeth. This work was soon followed by others, and at the commencement of the nineteenth century, the surgeon-dentists of this country were fully entitled to rank with the practitioners of the other branches of surgery.

"The most important of the works of our own time are those of Fox, 1803, Bell, 1829, Nasmyth, 1839, Owen, 1840; also those of Snell, Waite, Robertson, Jobson, and Koecker; besides which, we might enumerate several smaller works by Saunders, Clendon, White, and others, and many valuable detached papers in transactions and periodical publications." P. 13.

Now for the list of American authors whom Mr. Robinson arrays so triumphantly as evincing so great a superiority over those of his own country.

"Within the last century, dentistry has advanced far more rapidly in the United States than in any other country. Thus we have Gardette in 1821, Parmly,

L. S. Parmly, and Flagg in 1822, Trenor, 1828, Fitch, 1829, Brown, 1833, Spooner, 1836, Goddard, 1843; and in 1845, Dr. Harris, one of the editors of the *American Journal and Library of Dental Science*, published a most able and comprehensive work, entitled the *Principles and Practice of Dental Surgery*. And many other productions on the subject have appeared in America, and especially in the periodical just alluded to." P. 14.

Now really we could hardly help smiling at this extraordinary comparison; for we cannot but think that the list of English authors on "Dentistry," as here given by Mr. Robinson, from Hunter downwards, may possibly bear a comparison with his vaunted list of American writers, aye, even if we give them Mr. Robinson into the bargain.

The following farrago may be well quoted as the summing up of this comparative view of the merits of the practitioners of the two countries.

"Before concluding we may be allowed a word respecting the present state of dental art and science. The conditions of success appear to be not different in this from what they are in other branches of knowledge and practice. They are all summed up in one phrase, UNITED LABOURS. Whatever of discrepancy there is in the works of our chief authorities, is greatly owing to the isolation in which they studied, and to the want of a general means of collating their ideas. Again, whatever of progress we find in that country which takes the lead in the dental art, appears to be due to an absence of prejudice and jealousy which allows free communication of ideas, and association of common interests, among the members of the profession. For the association of dentists in America has not only given its members generally a *status* in society unknown to dentists elsewhere,—has not only repressed those characters who intrude themselves upon the public here, and given merit its station and honesty its preëminence,—but has also contributed largely to the advanced state in which dental science stands in the United States. It is painful to think that we do not yet possess the same advantages in England. The names of Harris, Brown, Parmly, Maynard, Greenwood, Goddard and Haydon, shine high over our heads in these respects, and present us with bright examples of brotherly good feeling, scientific excellence, and practical success." P. 16.

We fear that few of our readers have had the advantage of perusing the authorized medium of communicating information on "Dentism," (we use another of Mr. Robinson's Americanisms), and we must therefore ask them to take our word when we assure them that, as far as we can judge from that and other sources, we are patriotic enough to believe that we could select a body of practitioners of Dental Surgery in this metropolis, every one a member of the Royal College of Surgeons of England, who need not fear a comparison with those of any other country, no, not even of America, in knowledge of their profession, in the education, manners and feelings of gentlemen, in their "status in society," and in every other quality, which ought to distinguish the professional man or the gentleman.

Let us assure Mr. Robinson that we are sincerely pained at being forced upon this comparison; the attack is his own, and he must not feel hurt at our defence.

We proceed with our analysis. In the course of some very useful and judicious remarks on the diseases consequent on the irritation produced by the first dentition—and most of which, by the way, we seem to recollect to have met with in substance before—we have a very proper and rational denunciation of the common administration of opiates by nurses and mothers, concluding with this undoubted and important truth. "A teaspoonful of castor-oil will commonly be a far more efficient *opiate* than all the Godfrey's Cordials and Soothing Syrups that were ever invented."

In the observations on the "progress of the second or permanent teeth," we find nothing new worth transcribing; and the same may be said of the chapter on the second or permanent teeth, and that on the "Comparative View of the Teeth of Animals;" but the writer's views on the mechanical treatment of irregularity require some notice.* The general impression left by the perusal of this portion of the work is, that the author's prevailing tendency to the mere mechanical art of dental practice, has led him to depend more than is proper upon such means for remedying these cases. He appears to us to consider far too little the mischief which so often results from the application of mechanical force to growing teeth, and even to those which are already perfectly formed. At the same time we think that there are many practical suggestions which are likely, with proper caution, to be useful to the practitioner. He very properly condemns the common practice of extracting the temporary teeth "before the second are sufficiently developed to take their place." On this subject he relates a case, which does not appear essentially to differ from scores of almost daily occurrence; the only peculiarity, as far as we can see, being that the patient was "the son of a nobleman." (By the bye, what on earth does Mr. Robinson mean by "the pale of civilized dentism?")

On the subject of remedying irregularity arising from crowding of the teeth in the jaw from the arch being too narrow for them, a somewhat complicate apparatus is recommended (pp. 64-65), which appears to us to deserve a trial. There is, however, an obvious objection to Mr. Robinson's plan, and that is its being applied at as early an age as from the 9th to the 12th year. Not only is there great danger of loosening the teeth from their being moved at so early a period, but we should fear that the proper formation of the dentine would be interfered with, by the action of any considerable pressure on the growing tooth. Did Mr. Robinson ever examine the pulp cavity of a permanent tooth at this age?

We next pass on to the immobility of the jaw, arising from contraction of the elevators of the lower jaw, (we suppose this is what our author means); and we find this symptom, whether arising "from the cutting of the wisdom teeth, from the use of mercury, from hydrophobia, or *some* other cause (!)" all considered as "tetanus or lock-jaw!" The instrument recommended for forcing open the mouth in such cases is nothing more than the *speculum oris*, which is employed at every hospital, and sold by every instrument-maker.

On the subject of the "colour of the teeth as a test of consumption, &c.," our author seems to consider that, if people's teeth were examined, they would present such appearances, as would often lead to a plan of treatment by which phthisis would be prevented or cured. He appears to take great credit for his discovery of these new opinions; but we do not hesitate to say, that the effect of a scrofulous constitution on

---

* The following juxta-position of names strikes us as being funny. "Some curious anomalies of the teeth are related by *Pliny* and *Dr. Pritchard*."—Note, p. 36.

the appearance of the teeth has been known to every writer of consequence on the teeth, from Hunter to the present time; and many of the details which Mr. Robinson adduces, have, we are convinced, no existence but in his own imagination.—See particularly the coloured engraving at p. 78.

The subject of "the teeth considered as a test of age," is one of great importance, or, rather we might say, it would be so, were such indications to be depended on to any extent. We have ourselves seen so many examples of abnormal periods of the second dentition, that we entirely agree with Mr. Robinson, in considering such a test as absolutely fallacious. We have cases within our recollection, in which the teeth were nearly in the same state of progress at 5 and at 8, at 9 and at 13.

We come however to the *vexata questio*, the cause of caries. Here our author acknowledges himself at fault; and, after giving what he calls the opinions of about a dozen of writers on the subject, gives his verdict, without stating for what reason, for that originated by Parmly, and "maintained by Dr. Harris in his last work," (p. 88.) Now we beg to say, that the statements of Hunter, of Fox, of Bell, and of Saunders, are identical; and that the others agree only in attributing this disease to external causes alone. In other words, that the disease is attributed by the first-named writers to changes in living structure, in the others, to changes effected in the inorganic substance by which that structure is covered and protected. Mr. Robinson says,—"And now, having cited the opinions of others, I shall perhaps be expected to register my own. The field of speculation, however, is well enough occupied without it; and moreover, any view would be practically worthless, unless it enabled us to foresee the disorganization of the teeth, which we cannot do at present. I will nevertheless commit myself so far as to observe, that the nearest approach to truth appears to me to be the chemical theory of Parmly, put forth in the year 1820, and maintained by Dr. C. A. Harris in his last work." We are therefore at once enabled to judge what are the opinions of the author, of his Magnus Apollo Dr. Harris, and of the originator of the theory Dr. Parmly; and the passage to which we are referred as containing the enumeration of the decisive theory is the following precious sentence:—

"*Parmly* (1820): "The premature decay of the teeth, is the consequence of uncleanliness, which acts upon them in the same manner as on other parts, by sapping and corroding the vital energy, and thereby causing them to moulder away." P. 87.

Now we have no hesitation in saying that, if any one physiologist of the two hemispheres were asked the real simple meaning of this passage, he would acknowledge, and with a smile too, that he knows nothing at all about it. And this is the physiology on which American "Dentistry" is founded! Well may our author exclaim, "ne sutor ultra crepidam."

The class of persons for whom this work is written may be easily ascertained by the GLOSSARY which the author has thought it necessary to append to it. The work purports to treat on the *surgical* and *medical* treatment of the teeth. What sort of surgeons must they be who require an explanatory *glossary* of such terms as ABSORBED, ALKALINE, ALVEOLAR PROCESSES, CHRONIC, DIAGNOSIS, FEBRILE, MAXILLARY BONES (!) MUSCLE, &c. &c.?

But we feel that we are bestowing more space on this work than it deserves. It were easy to comment with painful severity on almost every page. The whole affair is obviously an attempt at making a book for an especial purpose. We had hoped that this was not the case when we commenced this review, but it is so painfully forced upon us that it is impossible any longer to conceal or to apologize for it.

The only portions of the book which are really free from the general censure, appear to be those which relate to purely mechanical matters, and with these, *we* have nothing to do. The Dentist may, and we doubt not will, find some useful hints on these subjects, but the result of our examination of the work, as far as it has respect to true dental surgery, is, that it is calculated only to perpetuate the unhallowed union which we have been deprecating, and to endeavour (it will, however, be fruitless) to exalt a mechanical art into the rank of a profession. Once more we call upon the educated *Surgeon*-Dentists of this country at least, to repudiate the mere mechanism of "Dentistry," to render themselves independent of the art of tooth-making, and to shew that there is a distinction between the scientific surgeon and the mere mechanic who, by administering to the vanity of a faded coquette, or even by subserving the better object of assisting impaired mastication, and thus becoming the humble adjuvant to the surgeon and physician, ought to be, and *must* be placed in a lower and a distinct class of professional society.

